# Rotational thromboelastometry in children presenting to an Australian major trauma centre: A retrospective cohort study

**DOI:** 10.1111/1742-6723.13939

**Published:** 2022-02-24

**Authors:** Shane George, Elizabeth Wake, Amy Sweeny, Don Campbell, James Winearls

**Affiliations:** ^1^ Department of Emergency Medicine Gold Coast University Hospital Gold Coast Queensland Australia; ^2^ Faculty of Medicine The University of Queensland Brisbane Queensland Australia; ^3^ School of Medicine Griffith University Gold Coast Queensland Australia; ^4^ Trauma Service Gold Coast University Hospital Gold Coast Queensland Australia; ^5^ Faculty of Medicine Bond University Gold Coast Queensland Australia; ^6^ Intensive Care Unit Gold Coast University Hospital Gold Coast Queensland Australia; ^7^ Intensive Care Unit St Andrew's War Memorial Hospital Brisbane Queensland Australia

**Keywords:** paediatric, trauma, viscoelastic haemostatic assay

## Abstract

**Objective:**

This retrospective cohort study aims to describe patterns of rotational thromboelastometry (ROTEM™) results in paediatric trauma following the implementation of a ROTEM‐guided critical bleeding algorithm and major haemorrhage protocol (MHP).

**Methods:**

This retrospective observational study was conducted in a tertiary trauma hospital in Queensland, Australia, where point‐of‐care ROTEM was introduced for paediatric patients in 2014. All children aged less than 18 years who had a ROTEM test during their presentation between January 2014 and December 2017 for a traumatic injury were included in the dataset. Other children with a record in the hospital's trauma registry in the same period were also screened for blood product usage. Data were collected for frequency of ROTEM testing, pathology and ROTEM results, blood product and antifibrinolytic use along with injury related data. Compliance with recommended treatment thresholds for detected coagulopathy was also reviewed.

**Results:**

A total of 1039 children were listed in the trauma registry, including 167 children having a ROTEM test for trauma. Factors significantly associated with having a ROTEM test were older age, higher injury severity score (ISS >12) and penetrating injury. A result exceeding a treatment threshold was returned for 122 (73.1%) of 167 children, with hyperfibrinolysis identified in 88 (52.6%) of 167 and hypofibrinogenaemia identified in 54 (32.3%) of 167. Adherence with the recommended treatments for those children where a treatment threshold was exceeded was low in this cohort.

**Conclusion:**

The use of ROTEM‐guided blood component replacement is an emerging practice in children for both traumatic and non‐traumatic bleeding. Targeted replacement of identified coagulation defects guided by rapid point‐of‐care testing is an emerging alternative to fixed‐ratio‐based protocols. Further research is required to validate treatment thresholds in the paediatric population and further investigate the clinical outcomes for patients as a result of early correction of trauma‐induced coagulopathy.

**Key findings**

- The use of ROTEM‐guided blood component replacement is an emerging practice in children for both traumatic and non‐traumatic bleeding.
- This cohort demonstrated high levels of fibrinolysis across all injury severities and across different regions of injury. This may represent different pathophysiological pathways or be due to different injury patterns in children compared to adults and is worthy of prospective investigation.
- Further research is required to validate thresholds for factor and blood product replacement in the children and evaluate clinical outcomes as a result of early correction of trauma‐induced coagulopathy.

## Introduction

Trauma causes 40% of child deaths in high income countries, with haemorrhage being a leading cause of death in this population.^1^ In addition to the primary insult causing haemorrhage, trauma‐induced coagulopathy (TIC) is recognised as playing a significant role in the mortality of trauma patients, with growing evidence for the phenomenon in the paediatric population.^2^ TIC is particularly notable in paediatric patients with traumatic brain injury.^2^

Major haemorrhage protocols (MHP) remain an important part of haemorrhage management, but they have been slower to appear in the paediatric literature.^3^ While their use within the paediatric population is increasing, they are frequently based upon the adult evidence. Strategies include ratio‐based transfusion, with ratios approaching 1:1:1 of packed red blood cells, fresh frozen plasma (FFP) and platelets; viscoelastic haemostatic assay (VHA) guided MHP or a hybrid MHP using both VHA and fixed ratio transfusion.

Over recent years, there has been an increasing interest in targeted transfusion guided by point‐of‐care VHA.^4^ VHA is performed on whole blood and provides information on clot initiation, amplification, strength and stability allowing differentiation of the individual's components contributing to clot kinetics to be assessed.^5^ The use of VHA to guide blood product transfusion in severe trauma is now endorsed by some international guidelines^6^ but these recommendations do not extend towards the paediatric population. This is reflected in the clinical setting, with a recent audit revealing institutions with access to VHA did not use them to guide management in more than 50% of paediatric cases.^7^ Part of the reason for this is concern around the validation of VHA use in paediatric trauma management, clinician familiarity and confidence with interpretation.^3^

Developing an evidence base for more targeted transfusion strategies within the paediatric setting has implications for effective management of TIC, reducing transfusion related complications for patients, and for improved rationing of scarce blood products. The use of VHA is not yet standard practice in the adult or paediatric trauma setting but has increasing popularity in many regions. In other areas of paediatric practice, particularly cardiac surgery, VHA‐guided transfusion is standard care in many centres.^8^ The Fibrinogen Early in Severe paediatric Trauma study (FEISTY Junior) is currently recruiting paediatric trauma patients in centres throughout Australia and aims to provide further evidence in this area.^9^

**Figure 1:**
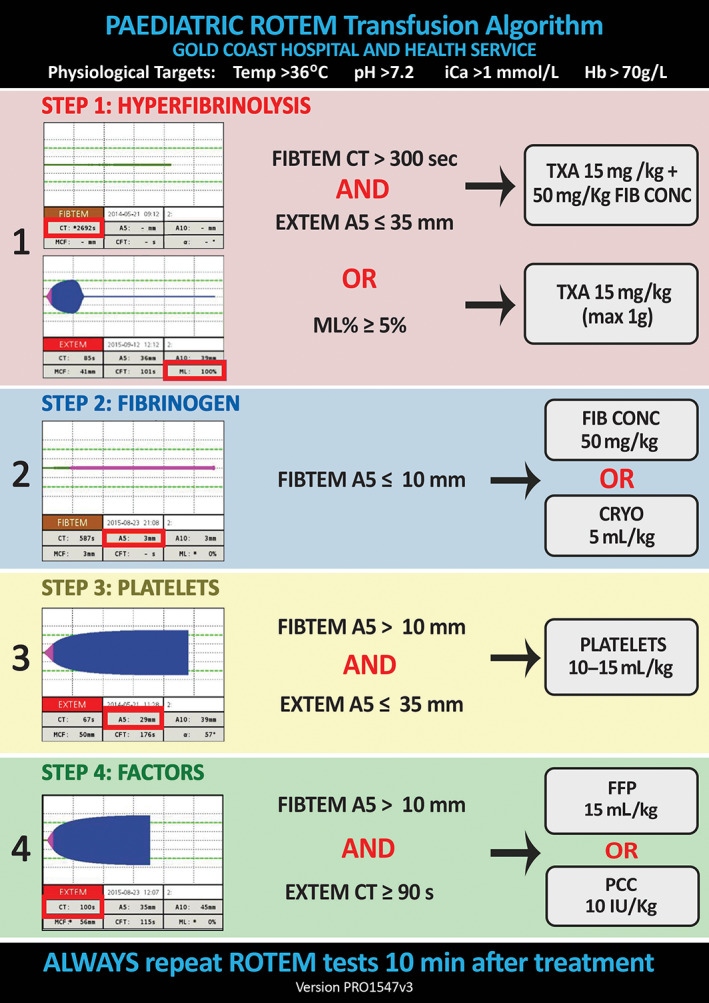
GCUH paediatric ROTEM^TM^ critical bleeding algorithm.

The Gold Coast University Hospital (GCUH) ED introduced a point‐of‐care rotational thromboelastometry (ROTEM™) into the critical bleeding algorithm for paediatric patients in 2014 (Fig. 1). ROTEM testing was initiated by the treating clinician with testing recommended in all children who met ‘Trauma Respond’ criteria (Fig. S1) and any other child with traumatic injuries where there was a clinical concern for bleeding (concealed or externalised) or significant injuries in conjunction with standard trauma pathology testing including laboratory based coagulation profiles.

The aim of this retrospective observational study was to review ROTEM results and the rates of targeted treatment for abnormal results in a paediatric trauma population following the implementation of a ROTEM‐guided critical bleeding algorithm. Data were analysed by injury severity and region of injury and compared to standard laboratory coagulation results.

## Methods

### Design and setting

This retrospective observational study was conducted at GCUH in Queensland, Australia. GCUH is a tertiary trauma referral hospital in Queensland, Australia, with over 1500 trauma presentations per year with approximately 350 (23.3%) being major trauma with an Injury Severity Score (ISS) of greater than 12. Over the study period, 1039 paediatric trauma presentations were recorded, with 103 scoring an ISS of greater than 12 (9.9%).

### Participants

All children aged less than 18 years old who received a ROTEM test during their presentation between January 2014 and December 2017 for a traumatic injury were screened for inclusion, those with traumatic injuries were included. Other children presenting during the time period with a record in the hospital's trauma registry were also screened for antifibrinolytic and blood product usage. All children who meet ‘Trauma Alert’ or ‘Trauma Respond’ criteria (Fig. S1) at triage are included in the hospital trauma registry. Children with ROTEM tests done for post‐partum haemorrhage, non‐traumatic bleeding, postoperative reasons (e.g. tonsillectomy related haemorrhage) or for snake bites were excluded.

### Data collection

Data were collected retrospectively from pre‐hospital, ED and inpatient records, laboratory records and the trauma registry. Data included, but were not limited to patient demographics, ISS, laboratory results from blood tests, and all blood and blood product usage (packed red blood cells, FFP, platelets, cryoprecipitate, fibrinogen concentrate, factor concentrate) during their hospital admission.

### Statistical analysis

Simple descriptive statistics (proportions) were produced for children who received ROTEM, and those from the trauma registry who did not. The χ^2^ test was used to determine significant differences between ROTEM testing groups, with a *P* < 0.05 considered statistically significant. ROTEM results were assessed according to the algorithm for each of the algorithm's four treatment thresholds (hyperfibrinolysis, hypofibrinogenemia, impaired clot formation, reduced clot strength); each child's results were classified as meeting or not meeting treatment threshold. ROTEM results were compared for children with more severe injuries (classified as ISS 12 or higher) to those with ISS <12. The first available blood results (haemoglobin, platelets, fibrinogen), collected after the child's triage date, were extracted from the laboratory information system. The *t*‐test was used to compare means of normally distributed variables and the non‐parametric Mann–Whitney *U*‐test was used to compare distributions of nonparametric variables. A Pearson product‐moment correlation coefficient was computed to assess the relationship between laboratory fibrinogen and ROTEM FIBTEM A5.

### Ethical considerations

This study was reviewed by the HREC committee and deemed a quality improvement initiative, not requiring ethics committee approval (LNR/2019/QGC/52798). The study is reported according to the STROBE guidelines.^10^

## Results

A total of 1039 children were listed in the trauma registry, including 167 children who had a ROTEM test for trauma during the period. Factors significantly associated with receiving a ROTEM test were older age, a higher ISS (ISS >12) and a penetrating injury (Table 1). Adolescents were more likely to have had a ROTEM performed with 87 (52.1%) of 167 patients in this sample aged 15–17 years. For all trauma presentations, regardless of ROTEM testing, the majority were transport‐related (57.4%), with a blunt mechanism of injury (Table 1).

**TABLE 1:** Characteristics of children presenting to the ED with trauma by whether or not they received ROTEM testing, Gold Coast University Hospital 2014–2017

	All presentations meeting trauma criterion (*n* = 1039)		ROTEM performed (*n* = 167)		No ROTEM performed (*n* = 872)		
	*n*	%	*n*	%	*n*	%	*P*‐value
Sex (female)	349	33.6	46	27.5	303	34.7	0.070
Age group (years)							<0.001
0–4	157	15.1	6	3.6	151	17.3	
5–9	261	25.1	25	15.0	236	27.1	
10–17	621	59.8	136	81.4	485	55.6	
Injury severity score							<0.001
<12	936	90.1	115	68.9	821	94.2	
12+	103	9.9	52	31.1	51	5.8	
Body region(s) of injury†							
Severe multi‐region injury	60	5.8	31	18.6	29	3.3	<0.001
Isolated severe head injury	42	4.0	11	6.6	31	3.6	0.086
Isolated severe chest or abdominal injury	57	5.5	17	10.2	40	4.6	0.007
Other severe isolated injury	26	2.5	12	7.2	14	1.6	<0.001
Mild/moderate injury(s) to other body part(s)	854	82.2	96	57.5	758	86.9	<0.001
Mechanism of injury							
Transport related	596	57.4	106	63.5	490	56.2	0.081
Fall	324	31.2	48	28.7	276	31.7	0.461
Struck by or against object or person	41	3.9	5	3.0	36	4.1	0.516
Cutting/piercing/stabbing	14	1.3	7	4.2	7	0.8	0.004
Fire/burn	14	1.3	1	0.6	13	1.5	0.399
Suffocation	8	0.8	0	0.0	8	0.9	0.243
Drowning	42	4	0	0.0	42	4.8	<0.001
Product administration							
RBC transfusion	26	2.5	22	13.2	4	0.5	<0.001
Massive RBC transfusion‡	5	0.5	5	3.0	0	0	<0.001
Fibrinogen (Cryo or FC)	19	1.8	18	10.8	1	0.1	<0.001
Fresh frozen plasma	7	0.7	7	4.2	0	0	<0.001
Platelets	7	0.7	7	4.2	0	0	<0.001
Tranexamic acid	27	2.6	27	16.2	0	0	<0.001

*P*‐values represent two‐by‐two χ^2^ mid‐*P* exact result for presence *versus* absence of each mutually exclusive characteristic *versus* ROTEM done, except for age group, which is a three‐by‐two comparison.

† Severe multi‐region injury = AIS >2 in one or more body regions with another injury AIS >1; isolated severe head injury = head injury with AIS >2 and no other AIS >1; isolated severe injury to chest or abdomen = AIS >2 for chest or abdomen with no other AIS >1; other isolated severe injury = AIS >2 for a single body region not otherwise defined, with no other AIS >1; mild/moderate injury(s) to other body part(s) = no individual AIS >2.

‡ Massive transfusion defined as greater than 40 mL/kg of packed red blood cells.

Most children with a ROTEM test had their first ROTEM test collected in ED (94.6%), particularly for those with less severe injuries (ISS <12) (Table 2). Most children had a single ROTEM performed (82.6%), although 24 (46.2%) of 52 children with a more severe injury (ISS >12) had at least one follow‐up ROTEM. ROTEM testing was performed a median of 37 min after triage (interquartile range 24.2–65.2 min).

**TABLE 2:** Description of ROTEM testing and results in 167 children following a trauma, by injury severity score (ISS)

Characteristic	All (*n* = 167)		ISS <12 (*n* = 115)		ISS 12+ (*n* = 52)		*P*‐value†
	*n*	%	*n*	%	*n*	%	
Place first ROTEM done							0.007
ED	158	94.6	113	98.3	45	86.5	
Intensive care unit/children's critical care unit	3	1.8	1	0.9	2	3.8	
Operating theatre	6	3.6	1	0.9	5	9.6	
Number of ROTEM tests per person							<0.001
1	138	82.6	109	94.8	28	53.8	
2	14	8.4	5	4.3	9	17.3	
3+	16	9.6	1	0.9	15	28.8	
Abnormality detected on first ROTEM‡							
None	45	26.9	36	31.3	9	17.3	0.079
Hyperfibrinolysis (ML% ≥5)	88	52.7	61	53.0	27	51.9	0.773
Hypofibrinogenaemia (FIBTEM A5 <10 mm)	54	32.3	31	27.0	23	44.2	0.026
Reduced clot strength (FIBTEM A5 >10 mm AND EXTEM A5 ≤35 mm)	3	1.8	2	1.7	1	1.9	Not calculated
Impaired clot initiation (FIBTEM A5 >10 mm AND EXTEM CT ≥90s)	1	0.6	0	0.0	1	1.9	Not calculated

† χ^2^ test ISS category *versus* characteristic.

‡ 31 children had two abnormalities detected at first ROTEM. None had more than two.

A result exceeding a treatment threshold was returned for 122 (73.1%) of 167 children, with hyperfibrinolysis identified in 88 (52.6%) of 167 and hypofibrinogenaemia identified in 54 (32.3%) of 167. Hypofibrinogenaemia was more likely in children with severe injuries (23/52, 44.2%) compared to less severe injuries (31/115, 26.9%, *P* = 0.026). Injury to multiple body regions and isolated head injuries were also more commonly associated with hypofibrinogenaemia when compared to mild/moderate injuries (*P* = 0.030 and 0.049 respectively, Table 3). There was no difference in observed rates of hyperfibrinolysis in severe injury (25/52, 48.1%) compared with less severe injury (61/115, 53.0%, *P* = 0.773). There were also no significant differences in detected hyperfibrinolysis when analysed by region(s) of body injury (*P* = 0.784, 4° of freedom, Table 3).

**TABLE 3:** ROTEM results by body region(s) of injury(s)†

	All with ROTEM (*n* = 167)		Severe multi‐region injury (*n* = 31)		Isolated severe head injury (*n* = 11)		Isolated severe chest or abdominal injury (*n* = 17)		Other severe isolated injury (*n* = 12)		Mild/moderate injury(s) to other body part(s) (*n* = 96)		
Characteristic	*n*	%	*n*	%	*n*	%	*n*	%	*n*	%	*n*	%	*P*‐value‡
Number of ROTEM tests per person													<0.001
1	137	82	18	58.1	6	54.5	9	52.9	10	83.3	94	97.9	
2	14	8.4	5	16.1	2	18.2	3	17.6	2	16.7	2	2.1	
3+	16	9.6	8	25.8	3	27.3	5	29.4	0	0.0	0	0.0	
Abnormality detected on first ROTEM§													
None	45	26.9	6	19.4	1	9.1	2	11.8	3	25.0	33	34.4	0.128
Hyperfibrinolysis (ML% ≥5)	88	52.7	15	48.4	7	63.6	11	64.7	6	50.0	49	51.0	0.784
Hypofibrinogenaemia (FIBTEM A5 <10 mm)	54	32.3	14	45.2	6	54.5	7	41.2	4	33.3	23	24.0	0.076
Reduced clot strength (FIBTEM A5 >10 mm AND EXTEM A5 ≤35 mm)	3	1.8	0	0.0	0	0.0	2	11.8	0	0.0	1	1.0	Not calculated
Impaired clot initiation (FIBTEM A5 >10 mm AND EXTEM CT ≥90s)	1	0.6	0	0.0	0	0.0	0	0.0	1	8.3	0	0.0	Not calculated

† Severe multi‐region injury = AIS >2 in one or more body regions with another injury AIS >1; isolated severe head injury = head injury with AIS >2 and no other AIS >1; isolated severe injury to chest or abdomen = AIS >2 for chest or abdomen with no other AIS >1; other isolated severe injury = AIS >2 for a single body region not otherwise defined, with no other AIS >1; mild/moderate injury(s) to other body part(s) = no individual AIS >2.

‡ χ^2^ test of characteristic by five body region(s) of injury(s) variable (three by five comparison (number ROTEM *versus* body region) or two by five χ^2^ (abnormality presence *versus* absence *versus* body region).

§ 31 children had two abnormalities detected at first ROTEM. None had more than two.

Adherence with the recommended treatments in the critical bleeding algorithm (Fig. 1) for those children where a treatment threshold was exceeded was low in this cohort. For 88 children who met the threshold for hyperfibrinolysis, administration of tranexamic acid (TXA) occurred in only 11 (12.5%); for 54 children meeting the fibrinogen treatment threshold, 14 (25.9%) received fibrinogen replacement; none of four children who met thresholds for either platelets or factors received any blood products. There were no children in the cohort who received products using the fixed ratio MHP.

In this cohort, only one patient was treated with fibrinogen replacement without having a ROTEM performed. There were no patients in the cohort who were treated with tranexamic acid without having a ROTEM performed. Of the 27 patients who received TXA, 11 patients were treated prior to having a ROTEM performed. Eleven of the remaining 16 patients received TXA directed by the ROTEM result and the remaining five patients were treated with TXA despite not meeting the treatment threshold.

In patients who received fibrinogen replacement, the majority (15/16, 93.8%) had a ROTEM performed before replacement. Similarly in those who received platelets or FFP, a ROTEM was performed prior to administration in all patients. There were no patients treated with platelets or FFP without a ROTEM being performed, unless they were administered prior to arrival to our centre, as part of an interhospital transfer.

**Figure 2:**
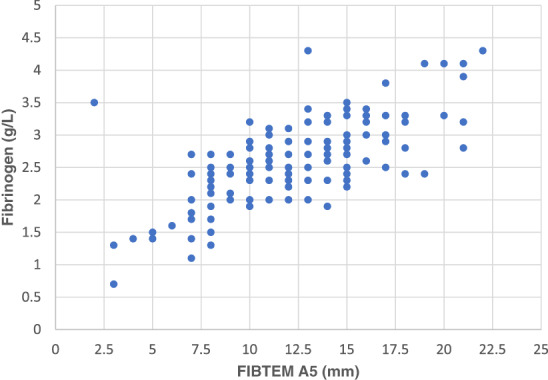
Correlation between ROTEM FIBTEM A5 and first fibrinogen result in 126 children presenting with trauma. Pearson's correlation coefficient = 0.680, *P* < 0.001.

Fibrinogen result by ROTEM (FIBTEM A5) was well correlated with the first fibrinogen result on a blood test (*r* = 0.68, *P* < 0.01, Fig. 2). While in general the results are well correlated, there were 13 patients with a laboratory fibrinogen of >2.0 g/L but a FIBTEM A5 of <10 mm and thus inconsistent treatment recommendations between testing methods in this cohort (Fig. 2).

## Discussion

The use of VHA in paediatric trauma and major haemorrhage is an emerging practice. Audits of paediatric practice in centres with access to VHA reported its use in less than 50% of paediatric cases.^7^ The reasons for this are likely to be multifactorial, with clinician familiarity with the tests and result interpretation along with the less frequent nature of paediatric traumatic haemorrhage playing an important role. In addition, while the normal values for both TEG and ROTEM have been reported in the literature^11^, ^12^ there is ongoing conjecture about what values should be used to guide treatment of abnormal results.^13^ Further research through prospective recruitment and patient‐centred outcomes are required to better inform clinical practice.

This cohort demonstrated high levels of fibrinolysis across all injury severities and across different regions of injury. This finding is consistent with previously published data reporting hyperfibrinolysis occurring more commonly in children when compared to adults.^14^ The reasons for this difference are unclear but may represent different pathophysiological pathways and mechanisms of injury in children compared to adults. The components of the fibrinolytic system are all present at birth; however, the ratios of key elements are different to those seen in adults. In particular, plasminogen and tissue‐type plasminogen activator (tPA) are significantly lower in children and adolescents when compared to adults, while plasminogen activator inhibitor‐1 (PAI‐1) levels are significantly elevated compared to adults.^15^

The exact incidence of hyperfibrinolysis in adult trauma remains elusive and has not been definitively established; however, the estimated frequency ranges from 3 to 20%.^16^ In the adult population, fibrinolysis of more than 3% has been associated increase in mortality and massive transfusion and is therefore considered the critical value for initiation of antifibrinolytic therapy.^17^ Similarly in children, there has been a reported increase in mortality once laboratory markers of fibrinolysis exceed 3%.^14^

The difference in incidence of hyperfibrinolysis is worthy for further prospective multisite data collection to exclude selection bias as a contributor to this observation. Further exploration of pathophysiological mechanisms that may further explain this observed difference between children and adults is also warranted to better understand the balance between physiological and pathological fibrinolysis and guide the development of treatment thresholds. In addition, studies into the empiric use of TXA in paediatric trauma focused on long‐term patient‐centred outcomes are warranted. Future prospective studies could investigate the roles of plasminogen, tPA and PAI‐1 in children, which may contribute to this finding.

Incidence and patterns of hypofibrinogenemia in this cohort are consistent with previously published data.^18^ The high incidence of hypofibrinogenemia in this relatively small cohort might suggest that there is a high rate of undetected hypofibrinogenemia in this population and validation in a sequential prospective cohort is warranted. The early treatment of hypofibrinogenemia in multi‐region trauma is increasingly recognised to reduce the requirement for massive transfusion and reduce mortality in adults; however, there are limited data in children.^3^, ^19^, ^20^ Future studies focused on patient‐centred functional outcomes are required to better inform clinical practice.

The role of hypofibrinogenemia and its treatment in isolated head injuries is controversial in both adults and children. In adult patients with isolated head injury, low fibrinogen on ROTEM has been described as an early indicator of severe injury and predictor of mortality.^21^ The role for replacement of fibrinogen in the management of severe brain injury remains unclear in both adults and children.^22^, ^23^

There was low compliance with suggested treatments detailed on the critical bleeding algorithm when a patient met the treatment threshold in this cohort. The decision to administer blood products to an individual patient is a multifactorial decision‐making process based on the immediate needs and clinical situation the clinician encounters, while optimal adherence to a protocol is theoretically ideal the protocol cannot always be applied in every situation. In our centre, evaluation for TIC by ROTEM will continue and be encouraged for children presenting with major trauma and where there are concerns for bleeding. Ongoing acquisition of data and gained experience may assist clinical decision making and guide specific antifibrinolytic and/or blood product therapy, minimising exposure to unnecessary products in injured children.

### Limitations

This retrospective review is limited by the data that was entered and available from the electronic systems available. Cross‐referencing records from multiple systems were undertaken to minimise missing or inaccurate data. Aspects of clinical care and clinician decisions regarding administration of blood components or treatment could not be determined from the written notes in most cases. Similarly, the presence or clinical suspicion of clinically relevant bleeding is often difficult to determine from retrospective review of the notes. Where possible pre‐hospital data on the administration of TXA and blood products have been included; however, prehospital records were not available for all patients. Changes in clinician familiarity with the MHP may have occurred over the recruitment period; however, no significant differences in patterns of product usage or compliance with the algorithm were detected. As a retrospective convenience sample, this cohort is not adequately powered to detect changes in clinical outcomes related to adherence to recommended treatment thresholds.

## Conclusion

The use of ROTEM‐guided blood component replacement is an emerging practice in children for both traumatic and non‐traumatic bleeding. Targeted replacement of identified coagulation defects guided by rapid point‐of‐care testing is an alternative approach to fixed‐ratio‐based protocols. In our centre, evaluation for TIC by ROTEM will continue and be encouraged for children presenting with major trauma and where there are concerns for bleeding. Ongoing acquisition of data and gained experience may assist clinical decision making and guide specific antifibrinolytic and/or blood product therapy, minimising exposure to unnecessary products in injured children. Further research is required to validate treatment thresholds in the paediatric population and further investigate the clinical outcomes for patients as a result of early correction of trauma‐induced coagulopathy.

## Supporting information


**Figure S1.** GCUH trauma alert and respond activation criteria.

## Data Availability

The data that support the findings of this study are available from the corresponding author upon reasonable request.
