# Supplementary figures and images for: Rotational thromboelastometry in children presenting to an Australian major trauma centre: A retrospective cohort study
Source: Emerg Med Australas. 2022 Feb 24;34(4):590–8. doi: 10.1111/1742-6723.13939 (PMC9542394; doi:10.1111/1742-6723.13939)

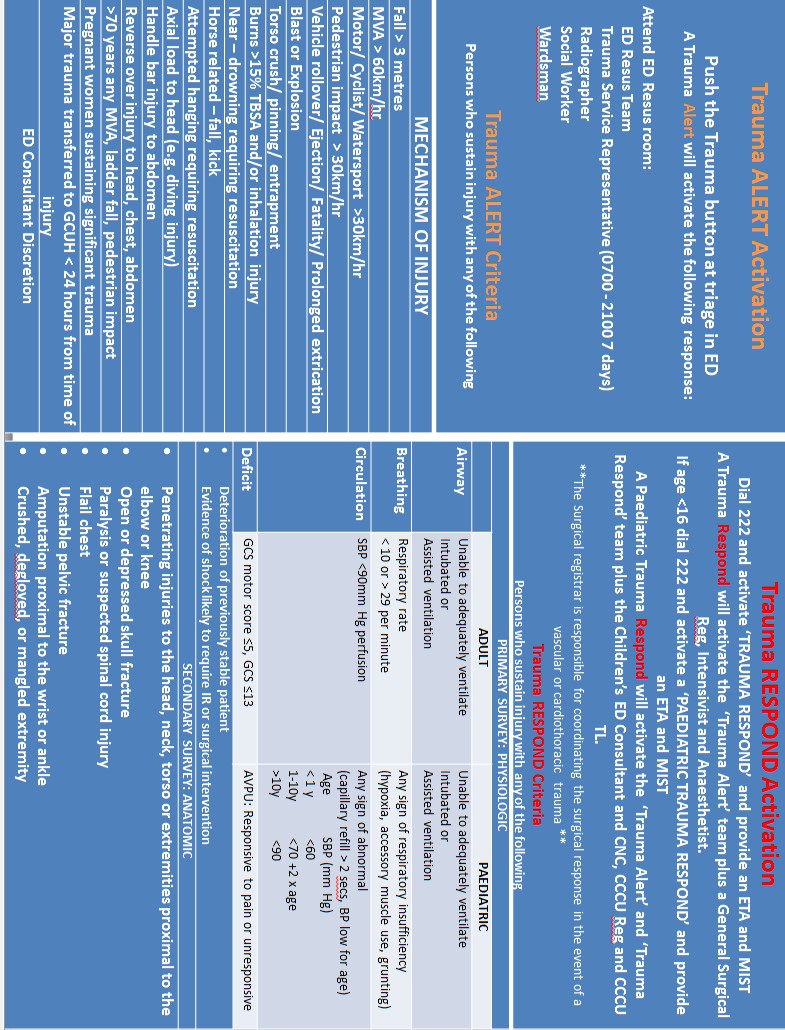
**Supplementary Figure 1: GCUH Trauma alert and respond activation criteria**

Supplement: Supplementary file 1 — Figure S1. GCUH trauma alert and respond activation criteria. [file EMM-34-590-s001.docx]
